# Localized Brain Activation Related to the Strength of Auditory Learning in a Parrot

**DOI:** 10.1371/journal.pone.0038803

**Published:** 2012-06-11

**Authors:** Hiroko Eda-Fujiwara, Takuya Imagawa, Masanori Matsushita, Yasushi Matsuda, Hiro-Aki Takeuchi, Ryohei Satoh, Aiko Watanabe, Matthijs A. Zandbergen, Kazuchika Manabe, Takashi Kawashima, Johan J. Bolhuis

**Affiliations:** 1 Department of Chemical & Biological Sciences, Japan Women’s University, Bunkyo-ku, Tokyo, Japan; 2 Japan Society for the Promotion of Science, Chiyoda-ku, Tokyo, Japan; 3 Department of Biology, Faculty of Science, Shizuoka University, Shizuoka, Japan; 4 Department of Physiology, Kitasato University School of Medicine, Kanagawa, Japan; 5 Behavioural Biology and Helmholtz Institute, Utrecht University, Utrecht, The Netherlands; 6 Graduate School of Social and Cultural Studies, Nihon University, Saitama, Japan; University of Rennes 1, France

## Abstract

Parrots and songbirds learn their vocalizations from a conspecific tutor, much like human infants acquire spoken language. Parrots can learn human words and it has been suggested that they can use them to communicate with humans. The caudomedial pallium in the parrot brain is homologous with that of songbirds, and analogous to the human auditory association cortex, involved in speech processing. Here we investigated neuronal activation, measured as expression of the protein product of the immediate early gene *ZENK*, in relation to auditory learning in the budgerigar (*Melopsittacus undulatus*), a parrot. Budgerigar males successfully learned to discriminate two Japanese words spoken by another male conspecific. Re-exposure to the two discriminanda led to increased neuronal activation in the caudomedial pallium, but not in the hippocampus, compared to untrained birds that were exposed to the same words, or were not exposed to words. Neuronal activation in the caudomedial pallium of the experimental birds was correlated significantly and positively with the percentage of correct responses in the discrimination task. These results suggest that in a parrot, the caudomedial pallium is involved in auditory learning. Thus, in parrots, songbirds and humans, analogous brain regions may contain the neural substrate for auditory learning and memory.

## Introduction

Darwin [1] already noticed the strong parallels between the acquisition of spoken language (speech) in human infants and song learning in songbirds [2]–[4]. In contemporary cognitive neuroscience, birdsong is a widely used animal model for human speech, because it is acquired through vocal imitation, thought to be an important prerequisite for the evolution of spoken language [5]. The capacity for vocal imitation is an evolutionarily rare trait, absent in non-human primates, but present in certain mammals and in three avian taxa, songbirds, parrots, and hummingbirds [5]–[10]. Thus, this capacity is unlikely to be the result of common ancestry, but rather a case of evolutionary convergence, whereby similar selection pressures were involved in solving similar problems in distantly related taxa [2], [11], [12]. Recent studies have shown that the behavioural parallels between birdsong and human speech can be extended to the neural [11], [13], [14], genetic [15]–[17] and possibly even the linguistic level [18], [19].

Parrots (of both sexes) can imitate vocalizations, including human words, throughout life [20]–[27]. In contrast, the most-studied songbird species, the zebra finch (*Taeniopygia guttata*), is an age-limited learner that does not acquire new vocalizations in adulthood [28]. The African grey parrot (*Psittacus erithacus*) can use imitated words to communicate with humans [29]. Parrots resemble humans also in that they use their tongue to articulate [30] and they can synchronize their movements to a musical beat [31]–[33]. Despite these intriguing behavioural parallels, there have been few studies investigating whether these similarities between humans and parrots are reflected in the neural mechanisms of auditory learning and memory.

Both parrots and songbirds have brain regions in the caudomedial pallium – the caudomedial nidopallium (NCM) and the caudomedial mesopallium (CMM) – that are analogous to the human auditory association cortex [2], [3], [13]. Within the songbird brain, Field L2 receives auditory connections from the thalamus and in turn projects onto Fields L1 and L3. These two regions project to the caudal mesopallium and caudal nidopallium, respectively. Similarly, the Field L2 of the budgerigar receives auditory input from the thalamus and constitutes the Field L complex with L1 and L3, which are thought to project onto the caudomedial pallium. This neuroanatomical similarity between songbirds and budgerigars suggests that the caudomedial pallium of the budgerigar may be functionally similar to that of songbirds.

Although the caudomedial pallium in songbirds has been found to be involved in song perception and memory [14], [34]–[43], we do not know whether these regions play a similar role in auditory learning and memory in parrots. To this end, we trained male budgerigars, a parrot species, in an auditory discrimination task involving two Japanese words produced by another male conspecific. We subsequently analyzed neuronal activation in the auditory forebrain of the males in response to re-exposure to the two discriminanda. Neuronal activation measured as the expression of the immediate early gene (IEG) *ZENK* has been very useful for mapping neuronal pathways activated through hearing song in songbirds (e.g. [44]) and the budgerigar [45]–[48]. Here we investigated the expression of the *ZENK* protein product Zenk in budgerigar males in response to exposure to the two discriminanda in an auditory discrimination task. We found stimulus-induced neuronal activation in the caudomedial pallium (particularly the NCM and the CMM) related to the strength of auditory learning. Our findings suggest that homologous brain regions are involved in auditory recognition memory in parrots and songbirds.

## Materials and Methods

### Subjects

All experimental procedures were in accordance with Japanese law and approved by the Animal Experiments Committee of Shizuoka University (Permit Number: 19-8, 20-6, 20-6-5) and by the Animal Experiments Committee of Japan Women’s University (Permit Number: 07-13). Eighteen adult male budgerigars were obtained from a local supplier. Each male was kept in an individual cage (32×26×39 cm) in the same room. All birds were maintained on a 14/10 hr light/dark cycle. Food and water were provided *ad libitum*, except during the auditory discrimination training period, during which food intake was restricted to maintain 80% of body weight before the start of training.

### Auditory Stimulus Recording

**Figure 1:**
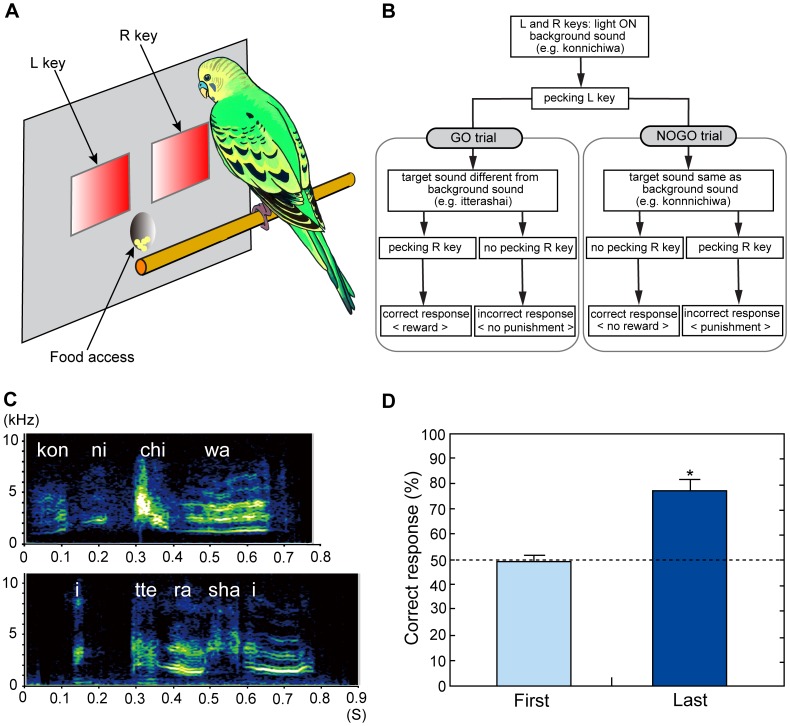
Budgerigars can discriminate Japanese words. **A** A schematic representation of the inside of the Skinner box. **B** Protocol of the go/no-go auditory discrimination task. **C** Sonagrams of the two Japanese words, spoken by a male budgerigar, used in the discrimination task. The top word means ‘hello’, and the bottom word means ‘have a nice day.’ **D** Mean proportion of correct responses in the go/no-go auditory discriminations. The mean (+ s.e.m.) percentage of correct responses for all of the trained birds over the first 5 sessions of training (100 trials per session) was not significantly above chance, but that over the last 5 sessions before stimulus re-exposure was significantly above chance (*n* = 7; ***p<0.005).

For use in the discrimination training and (re-)exposure sessions, we recorded two Japanese words, “*konnichiwa*” and “*itterashai*”, which were vocalized by a male budgerigar (Fig. 1C; Audio S1, Audio S2). This male budgerigar was unfamiliar to the tested birds. In listening to these two sounds, we could easily judge them as the imitations of Japanese words, although an earlier acoustic analysis has shown that budgerigar productions of human vowel sounds are not typical harmonic vocalizations as they are in humans [49]. The duration of *konnichiwa* was 690 ms, and that of *itterashai* was 660 ms. Both of these mimic sounds were recorded, using a digital audio taperecorder (SONY, TCD-D8, Japan) at a sampling rate of 44.1 kHz and a microphone (SONY, ECM-77).

### Apparatus

A Skinner box (29.5×30×21 cm) was placed in a sound-attenuating chamber (60×60×60 cm). Birds could peck two square response keys (3×3 cm) fitted into the rear wall (Fig. 1A). The response keys were equipped with red LCD lights. Pecking was detected with an infrared sensor set under a response key. Food as reinforcer was delivered to subjects through a food hopper, which was placed under the response keys. A wooden perch was placed in front of the food hopper. Auditory stimuli were played through a loudspeaker which was placed 30 cm behind the Skinner box, at 83 dB SPL peak amplitude measured at 30 cm away from the loudspeaker. During the auditory discrimination task, subjects were monitored via a video link on a screen (FUNAI, VC-N-141, Japan) outside the sound-attenuating chamber. We controlled the stimulus presentation, food hopper, pecking key lights and house lights with a personal computer.

### Training

We used an auditory discrimination task with a go/no-go operant conditioning procedure in [50]. Seven male budgerigars were trained to discriminate two different Japanese words originally produced by another budgerigar male (Fig. 1A,B,C). After a habituation period, the males were trained initially in a series of shaping routines using food rewards for pecks to response keys. For this task, we used the two words (*konnichiwa* and *itterashai*) mentioned above, as “background” and “target” sounds. Response keys on the left and on the right were turned on at the beginning of a trial of the auditory discrimination task. At the same time, one of the Japanese words (the background sound) was presented at the rate of once per second. During the presentation of the background sound, pecking the left key caused an auditory stimulus to switch from the background sound to the target sound (the other of the Japanese words) which was presented for 3 seconds (one sound per second). In order to switch from the background sound to the target sound, birds had to peck the left key, but not the right key, in all trials. There was a time limit (3 s) beyond which, if the bird failed to press the left key, the trial was terminated. Pecking the right key during the presentation of the background sound was punished by extinguishing all illumination for 5 seconds. Each bird was trained with four combinations of the background and the target sounds (that is, *konnichiwa* - *itterashai*, *konnichiwa* - *konnichiwa*, *itterashai* - *konnichiwa*, and *itterashai* - *itterashai*). In each trial, the computer selected the sound combination randomly from the four combinations, resulting in a counterbalanced presentation rate of each combination for a given subject. During presentation of the target sound that was different from the background sound (go stimulus), pecking the right key (correct go response) was food reinforced, while continued pecking of the left key caused training to proceed to the next trial. When the target and the background sounds were the same (no-go stimulus), continued pecking of the left key during the presentation of the target sound initiated the next trial (correct no-go response), while responding to the right key was punished. After the target sound started, each male had to either peck the right key (go trial), or withhold a peck to the right key (no-go trial) for the correct response in all trials. Each training session was finished when 100 trials were reached or after 30 min. Before test exposure, a mean of 57.9 sessions were completed for the 7 trained birds (range = 28–109 sessions). The trained birds did not mimic the Japanese words.

### Test Exposure

The trained birds were re-exposed to the discriminanda 2 to 5 days after the end of the last discrimination training session. During this time, all birds (7 Trained, 5 Untrained and 6 Silence) were kept in a room before the test day, where they could have auditory/visual contact with conspecific individuals, after which they were placed individually in a sound attenuated chamber and allowed to rest overnight. On the day of (re-)exposure, lights were switched on at 8:00 AM as usual. Subsequently, lights were switched off 15 min before the onset of playback, which started at 10:00 AM. Each bird was exposed to the two auditory stimuli (the Japanese words) used for the discrimination task, *konnichiwa* and *itterashai*, for 30 min. A series of *konnichiwa* and a series of *itterashai* were alternately played back at 83 dB SPL peak amplitude measured at 30 cm away from the loudspeaker (SONY SRS-A50, series duration, ca. 10 s; one sound per s; interval between series, 5 s). The birds remained in darkness for 1 hour after the end of (re-)exposure. Birds in group Silence received the same treatment throughout, except that they were kept in silence within a sound attenuation chamber. During playback, birds were kept in darkness to keep movement that could induce IEG expression to a minimum and to prevent the males from vocalizing [34], [51]. Pilot experiments showed that male budgerigars placed singly in chambers did not vocalize in darkness, as has been shown previously in zebra finch males [34]–[37], [41]. We monitored vocal behaviour of 12 birds (group Trained, *n* = 4; group Untrained, *n* = 4; group Silence, *n* = 4) during 30 min of playback and found that these birds did not vocalize. The audiotapes for the remaining six birds were lost because of technical problems. We conducted the same statistical analyses that we reported in Results section, using the 12 birds which were confirmed not to vocalize during playback. These additional analyses yielded qualitatively similar results.

### Immunocytochemistry

One hour after the end of exposure to the stimulus, the birds were anesthetized and subsequently perfused intra-cardially with saline and a Zamboni fixative (4% paraformaldehyde in 0.1 M PBS containing 10.5% of a saturated picric acid solution). The brains were post-fixed in the same fixative overnight and then stored for 1–2 days in a 30% phosphate-buffered sucrose solution at 4°C for cryoprotection. Frontal frozen brain sections were processed for Zenk expression by immunocytochemistry, as described previously [48]. Briefly, free-floating coronal sections (30 µm) were prepared and then sequentially incubated as follows: (i) 30 min in 3% H_2_O_2_ in methanol; (ii) 30 min in 0.2% Triton X-100 in PBS; (iii) 20 min in normal goat serum; and (iv) 48–72 h with the primary antibody (at 4°C). We used polyclonal antibodies against egr-1 (Santa Cruz Biotechnology, USA). To stain the sections, they were incubated (v) for 1 h in biotinylated goat anti-rabbit IgG, (vi) for 30 min in a streptoavidin–biotin–horseradish peroxidase complex, and finally (vii) for 30 min in diaminobenzidine and H_2_O_2_. Each of the steps above was followed by two or three washes in 0.01 M PBS. Brain sections were mounted on gelatine-coated slides, dehydrated in ethanol, cleared in xylene, and then cover-slipped with Entellan (Merck, Germany). Adjacent sections were Nissl stained to enable identification of anatomical markers. We visually inspected all the sections analyzed in our study to check for any failure in our staining procedure. ZENK gene expression was very low throughout the brains of quiet controls, except for a number of regions, including the region surrounding the lateral part of Field L2 (their figures 3N-P in [45]). In this region, there was consistent staining of Zenk-immunoreactive cells in all three treatment groups. This region is furthest from the brain’s surface compared with the regions analyzed in our study, and thus most vulnerable to insufficient fixation. Thus, it is unlikely that there were staining problems in our sections.

### Image Analysis

**Figure 2:**
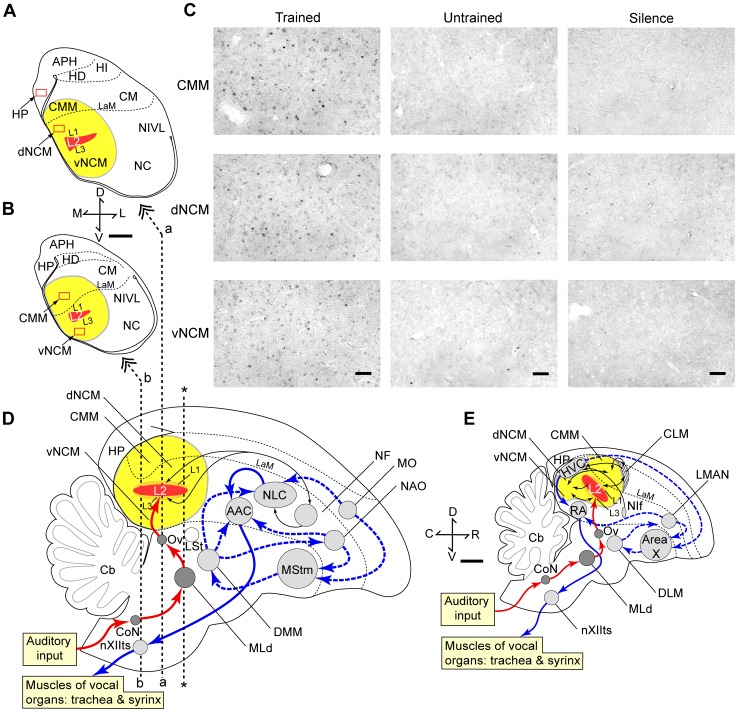
Neuronal activation in the brain. **A,B** Coronal sections of the budgerigar brain at the level of the dNCM and the hippocampus (A, cut at level “a” in D) and at the level of the vNCM and the CMM (B, cut at level “b” in D). Overlays represent the counting frames. Scale bar represents 1 mm. **C** Photomicrographs of coronal sections of the budgerigar brain showing Zenk immunoreactivity. Representative examples of Zenk-immunoreactive nuclei in the CMM (upper), the dNCM (middle), and the vNCM (lower) of birds that were trained and re-exposed to Japanese words (left), were not trained and exposed to Japanese words (middle), or kept in silence (right). Scale bar represents 50 µm. **D,E** Schematic diagrams of parasagittal views of the brains of avian vocal learners, parrots (D) and songbirds (E). Yellow regions indicate the caudomedial pallium, the NCM and the CMM. Ascending auditory pathways to Field L are similar in the two taxa (red arrows). Light grey regions indicate the vocal control system in parrots and the song system in songbirds. Lesion studies in adult and young songbirds led to the distinction between a caudal pathway (blue arrows), known as the song motor pathway (SMP), considered to be involved in the production of song, and a rostral pathway (blue dashed arrows), known as the anterior forebrain pathway (AFP), thought to play a role in song acquisition and auditory-vocal feedback processing. Equivalent pathways to the songbird SMP and AFP are proposed in the budgerigar [45], [79]. Scale bar represents 1 mm. AAC, Central nucleus of anterior acropallium; APH, Parahippocampal area; Cb, Cerebellum; CLM, Caudal lateral mesopallium; CM, Caudal mesopallium; CMM, Caudomedial mesopallium; DLM, medial nucleus of dorsolateral thalamus; DMM, Magnocellular nucleus of the dorsomedial thalamus; HD, Densocellular part of the hyperpallium; HI, Intercalated part of the hyperpallium; HP, Hippocampus; HVC, acronym used as a proper name; L1, L2, L3, subdivisions of Field L complex; LaM, Mesopallial lamina; LMAN, Lateral magnocellular nucleus of the anterior nidopallium; LSt, Lateral striatum; MO, Oval nucleus of mesopallium; MStm, Magnocellular part of medial striatum; NAO, Oval nucleus of the anterior nidopallium; NC, Caudal nidopallium; dNCM, Dorsal part of the caudomedial nidopallium; vNCM, Ventral part of the caudomedial nidopallium; NF, Frontal nidopallium; NIVL, Ventral lateral nidopallium; NLC, Central nucleus of the lateral nidopallium; nXIIts, tracheosyringeal portion of the hypoglossal nucleus; RA, Robust nucleus of the acropallium.

In songbirds, song-induced *ZENK* expression is found in the caudal nidopallium, which includes the NCM and Fields L1 and L3, surrounding a Zenk-negative L2 (Fig. 2E) [52]. Similarly, in budgerigars, Jarvis and Mello [45] found song-induced *ZENK* expression in the NCM and Fields L1 and L3 (Fig. 2D). As these authors could not distinguish Nissl boundaries between the different nidopallial fields outside of a Zenk-negative L2, they designated the entire region that showed hearing-induced expression in the nidopallium as the budgerigar NCM, which surrounds the presumed Fields L1 and L3. With Nissl staining, we also could not distinguish boundaries between the different nidopallial fields outside of L2, but could identify L2 and the mesopallial lamina (LaM), which is a distinct boundary between the CMM and the NCM. In a series of call stimulation studies in the budgerigar, the terms NCM and CMM were not used [46], [47]. However, a part of Field L as identified by Brauth et al. [46], [47] corresponds to the NCM as the term is used in the studies by Jarvis and Mello [45], Eda-Fujiwara et al. [48], and in the present paper. The NCM and the CMM are widely conserved among bird species [6], [53], [54]. We followed Jarvis and Mello’s [45] nomenclature in the present study. In previous reports two locations were sometimes sampled in the NCM, owing to its larger size [48], [55]. In the present study we sampled two regions within the NCM at dorsal and ventral levels (dNCM and vNCM) comparable with those in our previous paper [48] (Fig. 2A, B, D). In the songbird literature a distinction between ventral and dorsal NCM has been made as well [55] (Fig. 2E), but those do not correspond to the regions defined and analyzed in the present study. There are differences in the orientation of Field L2 across avian species, Field L2 having a more vertical orientation and CMM shifted more rostrally in songbirds than in parrots (Fig. 2D, E) [56]. The budgerigar vNCM corresponds to what is called NCM proper in songbirds, including dNCM and vNCM in the study by Mckenzie et al. [55], whereas the dNCM as defined here may correspond to a region rostrodorsal to Field L2 in songbirds. As a control, we also investigated Zenk expression in the hippocampus, because previously we have not found effects of song exposure in this region in male zebra finches [36] (see also [57]), female zebra finches [37] (but see also [58]), or female budgerigars [48].

We captured photomicrographs of the counting frames with a CCD camera and counted the number of Zenk-immunoreactive cell nuclei, “blind” as to the experimental history of the subjects. We took a total of four photomicrographs from both hemispheres (2 from the left and 2 from the right) per region (CMM, dNCM, vNCM, hippocampus) per subject. The rostro-caudal level of coronal section containing the rostral part of L2 and the most caudal part of the lateral striatum (LSt) was defined as coordinates zero (see Fig. 2D). For each region, the mean number of Zenk-immunoreactive cells was calculated for the dNCM and the hippocampus at 1.00 and 1.06 mm caudal to coordinates zero (level a in Fig. 2D), and for the vNCM and the CMM at 2.00 and 2.06 mm caudal to coordinates zero (level b in Fig. 2D). Image analysis was carried out semiautomatically with a PC-based system equipped with the KS400 version 3.0 software (Carl Zeiss Vision, Oberkochen, Germany). A program had previously been developed in KS400 to quantify immunoreactive cells, which is described in detail in a previous report [59]. The circular shape factor, optical density and mean nucleus size were determined for each region to optimize the selection specificity of immunoreactive cells. This program uses the circular shape factor to exclude artifacts that are above background-threshold level but are not nuclei. Mean nucleus size was determined by precisely measuring the circumference of 5 nuclei per picture, in 5 random pictures. This measure is used by the program to exclude artifacts that are much smaller or larger than an average nucleus. We ensured accuracy of our cells counts by checking each selection of immunoreactive neurons made by the program, and deselecting artifacts manually. To facilitate this process, the selection of what is background staining (based on pixel intensity levels) could be adjusted when necessary for each picture independently.

### Statistics

IEG expression was analyzed with a repeated-measures analysis of variance (ANOVA) with Group (Trained, Untrained or Silence) as between-subject factor and Brain Region (dNCM, vNCM, CMM or hippocampus) as within-subject factor. Subsequently, we used one-way ANOVAs for individual brain regions. Post-hoc comparisons were done using Tukey-Kramer tests. Performance in the discrimination task was compared to the chance level using one-sample *t* tests (two-tailed). The relationship between the strength of learning (measured as the mean percentage correct in the last 5 discrimination training sessions) and the number of Zenk-immunoreactive cells per mm^2^ was examined using a Pearson’s product-moment correlation analysis. Raw data were log-transformed for the cell counts or arcsine-transformed for the percentages of correct responses to satisfy assumptions of the parametric tests [60]. Levels of significance were set at *p*<0.05. Data were analyzed using StatView version 5 (SAS Institute Inc.).

## Results

For the seven males trained in the auditory discrimination task, the mean value of the percentage of correct responses over the last 5 training sessions before re-exposure was 77.5±4.5% (mean ± s.e.m.), significantly greater than chance (*t*_6_ = 5.39, *p* = 0.002; Fig. 1D).

**Figure 3:**
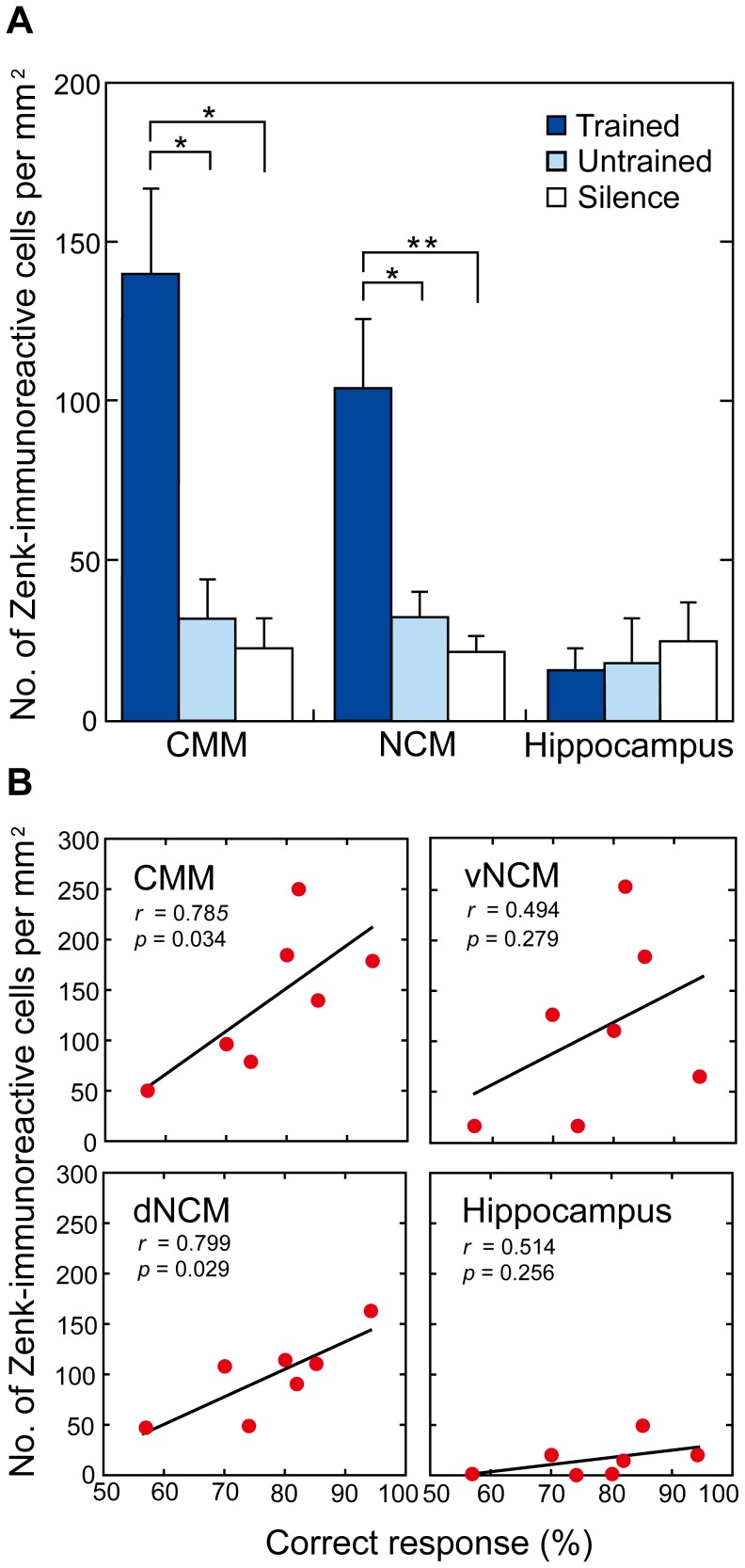
Neuronal activation related to the strength of auditory learning. **A** Mean (+ s.e.m.) number of Zenk-immunoreactive cells per square millimetre in the CMM, the NCM and the hippocampus for groups of male budgerigars in the Trained (*n* = 7), Untrained (*n* = 5) and Silence (*n* = 6) groups. Asterisks denote significant differences between the mean of the Trained group and the means of the two other groups (*p<0.05, **p<0.01). **B** Number of Zenk-immunoreactive cells per square millimetre in relation to the percentage correct responses in the discrimination task, in the CMM, the dNCM, the vNCM and the hippocampus. The correlation is significant in the CMM and the dNCM, but not in the vNCM or in the hippocampus.

In response to playbacks of the two discriminanda, we found stimulus-induced expression of Zenk in the NCM and the CMM (Fig. 2A–D). The mean number of Zenk-immunoreactive cells per square millimetre was calculated in the dorsal NCM (dNCM), the ventral NCM (vNCM), the CMM, and the hippocampus (Fig. 2A–D). An repeated-measures ANOVA revealed a significant effect of Brain Region on the number of Zenk-immunoreactive cells (*F*_3_,45 = 5.51, *p* = 0.003). Post-hoc tests revealed no significant difference in levels of Zenk expression between the dNCM and the vNCM. Thus, we used the mean value of the dNCM and the vNCM for the NCM. Because there was a significant effect of Brain Region, the results were analyzed for the different brain regions (CMM, NCM and hippocampus) separately. ANOVAs revealed a significant effect of Group (Trained, Untrained or Silence) on the number of Zenk-immunoreactive cells for the CMM (*F*_2_, 15 = 6.48, *p* = 0.009) and for the NCM (*F*_2_, 15 = 9.49, *p* = 0.002), but not for the hippocampus (*F*_2_, 15 = 0.76, *p* = 0.486; Fig. 3A). In both the CMM and the NCM, there was a significantly increased number of Zenk-immunoreactive cells in the Trained group compared to both the Untrained (CMM, *p*<0.05; NCM, *p*<0.05) and the Silence groups (CMM, *p*<0.05; NCM, *p*<0.01), but there were no such differences between the Untrained and the Silence groups (Fig. 3A). Thus, male budgerigars showed significantly increased neuronal activation in the CMM and the NCM, when re-exposed to the two auditory stimuli. Although the mean number of immunoreactive cells in the dorsal and ventral components of the NCM did not differ significantly, we performed one-way ANOVAs on the results for these two regions separately, in line with a previous study [48]. We found a significant effect of Group on the number of Zenk-immunoreactive cells for the dNCM (*F*_2_, 15 = 6.67, *p* = 0.009), but not for the vNCM (*F*_2_, 15 = 3.13, *p* = 0.073). In the dNCM, there was a significant difference in the number of Zenk-immunoreactive cells between the Trained group and both the Untrained (*p*<0.05) and the Silence group (*p*<0.05), but there were no such differences between the Untrained and the Silence groups.

The strength of auditory discrimination learning (expressed as the percentage of correct responses) was significantly positively correlated with neuronal activation in response to the two discriminanda (expressed as the number of Zenk-immunoreactive cells) in the CMM (*r* = 0.785, *p* = 0.034; Fig. 3B), but not in the NCM (*r* = 0.643, *p* = 0.127) or in the hippocampus (*r* = 0.514, *p* = 0.256; Fig. 3B).We performed subsequent correlation analyses on the two subregions of the NCM. There was a significant positive correlation between the number of Zenk-immunoreactive cells and the strength of discrimination learning in the dNCM (*r* = 0.799, *p* = 0.029; Fig. 3B), but not in the vNCM (*r* = 0.494, *p* = 0.279; Fig. 3B). There was no significant correlation between the number of Zenk-immunoreactive cells and the number of training sessions for any of the sample regions (CMM: *r* = 0.097, *p* = 0.846; dNCM: *r* = 0.150, *p* = 0.763; vNCM: *r* = −0.285, *p* = 0.558; hippocampus: *r* = −0.120, *p* = 0.806).

## Discussion

The present study shows that in the budgerigar, a parrot species, there is stimulus-induced molecular neuronal activation in the caudomedial pallium (particularly the NCM and the CMM) related to the strength of auditory learning. These findings suggest that the caudomedial pallium of a parrot is involved in auditory learning and memory, as it is in songbirds [2].

Jarvis et al. [61] argued that in studies analyzing the relation between IEG expression and learning it is essential to evaluate the contribution of factors such as stimulus novelty, stress and attention. Stimulus novelty has been found to lead to an increase in IEG expression in the NCM (songbirds: [61], [62], parrots: [46]). In contrast, in the present study, the stimulus words which were not novel stimuli lead to increased neuronal activation in the NCM and the CMM of budgerigars of the Trained group. Thus, it is unlikely that the increased neuronal activation in theses brain regions that we observed in the Trained group is related to stimulus novelty. Alternatively, it may be that the increased neuronal activation in the Trained group is explained by familiarity due to previous exposure to the sounds in these birds. In the present study, birds without previous exposure to the sounds would pay attention to playback sounds and might show stress responses. Stress responses in the Untrained and Silent groups might inhibit gene expression driven by auditory stimuli. If this is the case, within the Trained group, birds that experienced fewer training sessions might show lower IEG expression. However, we did not find such a correlation in the Trained group. Furthermore, pilot experiments showed that in male budgerigars similar to those in the Untrained group, Zenk expression in the NCM was high when birds heard novel conspecific vocalizations, as has been shown previously in female budgerigars [48]. Thus, it is unlikely that stress responses in the Untrained group inhibited gene expression driven by auditory stimuli. Whether IEG expression is a reflection of ‘predisposed’ attentional mechanisms [63] could be tested by exposing trained subjects to novel stimuli, which would show similar correlations. Terpstra et al. [36] did this for zebra finches and did not find such correlations for groups of males exposed to either novel song or the bird’s own song (BOS), only when exposed to tutor song. The present findings are consistent with the suggestion that the neuronal activation in the Trained group is related to auditory learning.

The birds in the Untrained group were exposed to the novel stimuli. Nevertheless, there was no difference in neuronal activation in the NCM between this group and the Silence group. Previous IEG studies in songbirds have shown that IEG expression in the NCM is high when the birds hear conspecific songs, significantly lower when they are exposed to heterospecific songs, and virtually absent during exposure to tone bursts [57], [58]. In male and female budgerigars, Zenk expression in the NCM is high when birds hear novel conspecific vocalizations [46], [48]. For the Untrained males in the present study, the stimulus Japanese words were novel heterospecific sounds, which may explain the low level of responsiveness in these birds.

The budgerigar males successfully mastered the auditory discrimination task. Performance in this task could be the result of at least two factors, or a combination of the two. First, it may be that the birds learned the association between stimulus change and reward. That is, they may have learned to respond appropriately upon detection of a salient change in a repeated auditory event (i.e., a change from one Japanese word to the other Japanese word). During testing, the birds were also exposed to alternating presentations of the two Japanese words. It is possible that the acquired association between stimulus change and food reinforcement drove IEG expression in the caudomedial pallium. Second, it may be that variation in performance in the discrimination task reflected the strength of auditory recognition memory. That is, successful performance may be due to the animals having formed a memory of the two auditory stimuli, and recognizing them in the discrimination task. Neuronal activation during re-exposure would then reflect activation of the representation of the two auditory stimuli, rather than a detection of stimulus change. Finally, it is possible that both these processes were at work during discrimination training and subsequent re-exposure.

The second interpretation, in which increased IEG expression in the caudomedial pallium would reflect activation of the representation of the memory of the two words, is supported by a previous IEG study in the budgerigar [46]. Repeated exposure to a conspecific call led to a waning of the IEG response in the budgerigar NCM [46], consistent with a possible role for this region in the detection of stimulus familiarity, which is an important aspect of recognition memory [64]. Furthermore, the neural similarities between songbirds and budgerigars suggest that the caudomedial pallium of the latter may have similar functions to that of the former. In line with this, the caudomedial pallium of both taxa shows neuronal activation related to song perception [45], [48], [57]. Similarly, the present findings are consistent with the suggestion that neuronal activation in the caudomedial pallium is related to the strength of auditory memory, just as it is in zebra finches [34]–[36], [41].

The present findings suggest a role of the CMM as well as the NCM in auditory memory in male budgerigars. Bolhuis et al. [34] found increased neuronal activation in the NCM and the CMM in zebra finch males exposed to tutor song, compared to silence. However, in that and a series of subsequent studies, significant correlations between the strength of song learning and IEG expression were found in the NCM, but not in the CMM, of male zebra finches [34]–[36], [41]. In zebra finch females there was significantly increased IEG expression in subjects exposed to their father’s song, compared to novel song, only in the CMM, not in the NCM [37]. Hernandez et al. [65] suggested that there are species differences in memory-related responsiveness in the caudomedial pallium in female songbirds. Electrophysiological studies in European starlings trained in an operant task, revealed memory-related responsiveness in the CMM and the NCM of males and females [66], [67]. Bolhuis and Gahr [14] suggested that the differential memory-related responsiveness of the CMM and the NCM observed in the zebra finch may be related to the sex of the birds. These authors argued that recognition of the father’s song is important in both sexes [68], and the CMM might contain the neural substrate subserving memory of that song. In many songbird species (such as the zebra finch) only males produce song, based on a memory of the tutor song, for which the NCM might contain the neural substrate, possibly serving as a parallel store to the CMM. In species where both males and females sing, such as the European starling and the budgerigar, memory-related neuronal activation would be expected to occur in both of these caudomedial regions, as is the case in the present study.

In another avian learning paradigm, filial imprinting. the medial nidopallium has been suggested to be involved in auditory imprinting [69], while neuronal activation in the intermediate and medial mesopallium (IMM) has been shown to correlate with the strength of visual imprinting in chicks [70]. Thus, the medial pallium may be generally involved in auditory and visual learning and memory in parrots, songbirds and other avian groups [14].

In songbirds there is continual interaction between the nuclei in the song system and regions in the caudomedial pallium. The forebrain nucleus HVC of songbirds is a nucleus of the song system. Bauer et al. [71] reported on auditory and singing-related activity of a region within the caudal mesopallium (CM). They also showed that inactivating this region shut down auditory activity in the HVC, demonstrating a functional connection between this region and the HVC. In addition, Akutagawa and Konishi [72] reported the projection from the HVC onto the region that Bauer et al. [71] had studied, but identified the region studied by Bauer et al. [71] as the avalanche nucleus (Av), a nucleus of the song system. The budgerigar vocal control (or motor) system [45], [46], [73], [74] is a network of interconnected nuclei similar but not identical to the song system of songbirds (Fig. 2D). The budgerigar nucleus NLC corresponds with the songbird HVC [75], [76]. Is there interaction between the caudomedial pallium and the vocal control nucleus NLC in the budgerigar? Results by Farabaugh and Wild (their figure 2) [77] suggest that in the budgerigar, in addition to Field L1, the dNCM projects to a region within the frontal nidopallium (NF), the term of which was revised to the lateral nucleus of the anterior nidopallium (LAN) [51]. This region in turn projects to the NLC (Fig. 2D) [74]. Thus, in parrots there may also be a functional connection between the caudomedial pallium and the vocal control system.

In summary, these results suggest that auditory discrimination learning by parrots involves regions in the caudomedial pallium, the avian equivalent of the human auditory association cortex. Homologous brain regions in songbirds have been found to contain the neural substrate for tutor song memory. Thus, in parrots, songbirds and humans, similar brain regions appear to be involved in auditory learning and memory. These brain regions can be homologous [11], [13], [78], but the function of such regions can be the result of convergent evolution. Thus, songbirds, parrots, rats, apes and humans may have analogous or homologous ‘auditory association cortex’-like regions, but only humans, parrots and songbirds show auditory-vocal imitation learning. Thus, our findings suggest that neural similarities (that may be homologous) are matched by evolutionary convergence at the cognitive level in parrots, songbirds and humans.

## Supporting Information

Audio S1 **The Japanese word, “konnichiwa”, vocalized by a male budgerigar.** It was used in the discrimination training and (re-)exposure sessions.
(M4A)

Audio S2 **The Japanese word, “itterashai”, vocalized by a male budgerigar.** It was used in the discrimination training and (re-)exposure sessions.
(M4A)
